# Supplementary material for: Influences on policy-formulation, decision-making, organisation and management for maternal, newborn and child health in Bangladesh, Ethiopia, Malawi and Uganda: The roles and legitimacy of a multi-country network
Source: PLOS Glob Public Health. 2023 Nov 21;3(11):e0001742. doi: 10.1371/journal.pgph.0001742 (PMC10662733; doi:10.1371/journal.pgph.0001742)
Supplement: Attachment — Submitted filename: Response to reviewers.docx [file pgph.0001742.s005.docx]

Dear Editors,

We thank the reviewers for their comments and have responded in blue text to each of their comments.

**Comments from the reviewers:**

**Reviewer #1:**

I applaud the authors for this carefully prepared paper. I am happy to recommend minor revisions to this paper, as it is already well thought out and written.

The premise that QCN needs legitimacy to work is one that is sound. As the authors write, “for the network to work as intended the government of each member country must buy into the idea and spend time and resource on network activities and coordination” (p. 3, line 57). To study these interactions, the authors draw on a wide range of perspectives on legitimacy to explain different degrees of buy-in to QCN by national and local policymakers in the four countries they study (of Bangladesh, Ethiopia, Malawi, and Uganda, as well as at the global level). The analysis focuses on “political, normative and cognitive interactions between institutions involved in QCN in each country” (p. 9, line 146).

Author’s response: Thanks very much for your detailed review of our paper.

1. I am writing here from a different disciplinary background as the authors, so please take these literature suggestions with a grain of salt and use as you see fit—we also don’t typically talk about legitimacy, but we do think in terms of trust. Here work from the sociology of global health may be useful in reframing some of the questions that the authors have (Harris and White 2019; Robinson 2017). These approaches generally, like the authors, take into account the institutional contexts and histories of the countries under study, but attempts to link through sequencing and looking at changes in “field configurations”, how actors build on previous meanings attributed to certain public health interventions (e.g. in Robinson, HIV/AIDS interventions are mapped onto the family planning field that preceded it). Drawing from this literature can also help link the material provided in the background sections to the empirical findings of the “present”. Potentially relevant also is the emerging literature from economic sociology on network failures, which also sees trust and legitimacy (as well as competence and opportunism) as key to the success of networks such as QCN, as well as other public-private partnerships, international organizations, supply chains etc. (Schrank and Whitford 2011), as well as with works in the sociology of development that looks at “pockets of efficiency” within large bureaucracies and the “reactive diffusion” of global networks with values, norms, priorities, etc. (Chorev 2019; McDonnell 2020).

References:
Chorev, Nitsan. 2019. Give and Take: Developmental Foreign Aid and the Pharmaceutical Industry in East Africa. Princeton University Press. <https://press.princeton.edu/books/hardcover/9780691197852/give-and-take>.
Harris, Joseph, and Alexandre White. 2019. “The Sociology of Global Health: A Literature Review.” Sociology of Development 5 (1): 9–30. <https://doi.org/10.1525/sod.2019.5.1.9>.
McDonnell, Erin Metz. 2020. Patchwork Leviathan: Pockets of Bureaucratic Effectiveness in Developing States. Princeton University Press. <https://press.princeton.edu/books/hardcover/9780691197357/patchwork-leviathan>.
Robinson, Rachel Sullivan. 2017. Intimate Interventions in Global Health: Family Planning and HIV Prevention in Sub-Saharan Africa. Cambridge: Cambridge University Press. <https://doi.org/10.1017/9781316117033>.
Schrank, Andrew, and Josh Whitford. 2011. “The Anatomy of Network Failure.” Sociological Theory 29 (3): 151–77. <https://doi.org/10.1111/j.1467-9558.2011.01392.x>.

Author’s response: Thanks very much for these useful suggestions. We have added reference to this literature in our introduction and our discussion sections.

We especially found it useful to highlight the emphasis in the sociology scholarship on institutional contests and histories of the involved countries in considering an initiative’s nature and perceived legitimacy. We have significantly revised the third paragraph of the introduction to include relevant scholarship from sociology that the reviewer recommended:

“Scholarship in sociology emphasizes consideration of institutional contexts and/or histories of the involved countries in considering an initiative’s outcomes and perceived legitimacy (Shrank and Whitford 2011; Harris and White 2019). For example, Robinson (2017) illustrates how preceding experiences in family planning prefigured—and directly impacted the perceived legitimacy and nature of—national HIV prevention strategies in sub-Saharan Africa.”

We also then made reference to this literature and others in the discussion, in illustrating that our paper’s findings confirm and build upon ideas from previous scholarship in international studies and sociology:

“In addition, the findings support and build on scholarship in international studies and sociology, which find legitimacy to be influenced by a network’s mission and value compatibility with local contexts, as well as a country’s history.”

2. The comparative framework, research design, and methods is also sound. One of the methodological problems that other studies of this sort have is to assume that “legitimacy” is a thing that is out there to be measured and captured—that respondents either believe there is legitimacy, or not, or possess it to a certain degree that can be quantified. Instead, the authors focus on social processes that construct legitimacy.

The authors are able to use iterative interviews from 2019 to 2022 to trace these processes. The methods are also described in Supplement 2, but it would be helpful to give a quick gesture to the number of interviews, length of interviews, etc. in the main text so readers can more easily locate this information. The supplements are also very detailed, and I applaud the authors for the care they took to document the data and methods they employed. The author’s request to withhold interview data should also be granted—as is the norm for qualitative interviews of this sort of potentially identifiable respondents.

Author’s response: Thanks very much, and for this useful suggestion. We have added information on the number and length of the interviews to the methods section. We agree that the information in the interviews could identify respondents, so we are not able to make the interview data freely available.

3. The analysis and results sections are presented first by providing necessary background into the four cases, and then looking at the political, normative, and cognitive interactions that create legitimacy. Illustrative quotes are presented to highlight these interactions at work. And the discussion section goes over these more broadly and summarizes the presence/absence of these interactions in the four cases. One question I have with this section is that it seems like the authors are taking an “additive approach” to legitimacy: Legitimacy is calculated or qualitatively described by adding up the presence of all these different types of interactions. But are there types of interactions that lead to less legitimacy? For instance, if actors learn of information (cognitive interactions) that is interpreted as negative or bad, does this detract from legitimacy of QCN? Some clarification would be helpful here.

Author’s response: This is a good point. There are multiple ways to conceptualize legitimacy and we could have looked at things that take away from legitimacy. We didn’t find things that take away from legitimacy in our data though – rather just presence or absence of positive things. We recognize this as a limitation and have added the following sentence to the penultimate paragraph in our discussion section:
“We did not find evidence of interactions that led to less legitimacy of the network, hence our focus on the presence or absence of interactions that lead to greater legitimacy of the network.”

4. The conclusion that “we find evidence supporting the legitimacy of QCN from the perspective of country governments, further work and time are required for governments to own and embed the work of QCN in routine care” is also sound. But it would be helpful for the authors to develop this point a little bit further and develop specific recommendations on how QCN can be incorporated in routine care. Here the authors should also address how legitimacy is different when implemented at the micro-level, in clinical settings, etc.

Author’s response: Thanks for this suggestion. This paper is focused on global and national levels of the network. The micro-level of care, and specific recommendations on how QCN can be incorporated into routine care, are beyond the scope of this paper and is the focus of other papers in our collection of papers evaluating the network including Djellouli *et al* on network effectiveness (reference 11). We believe the last paragraph of our discussion on future directions for research (now with new second sentence added) covers these issues:

“The findings from this paper are useful as context for our assessments of the effectiveness of the network in delivering interventions and changing processes of care [11] and in understanding how it operates [33]. In addition, the findings support and build on scholarship in international studies and sociology, which find legitimacy to be influenced by a network’s mission and value compatibility with local contexts, as well as a country’s history [4-7]. Further research looking at legitimacy and ownership of the work of QCN, and networks of care more broadly [32], at district, health facility and community levels within countries will be useful to deepen understanding of what drives networks and how best to embed their work into routine systems and sustain them.”

5. On p. 2, line 32 (and subsequent uses of this phrase): “We developed a framework drawing on three frameworks”, perhaps change one of the frameworks to perspectives? Or some other synonym.

Author’s response: Thanks, we have changed the second “framework” to “model” and now refer to the three frameworks of Tallberg and Zurn, Nasiritousi and Faber, and Sanderink and Nasiritousi, as “models” accordingly, throughout the paper.

6. On p. 28, line 561: “We found political interactions to be good in all four case study countries…”, perhaps replace “good” here with something referring to the frequency and quality of these interactions? “Good” sounds like you are judging the outcomes of these interactions here.

Author’s response: We replaced the word ‘good’ with ‘strong’. Thanks so much.

**Reviewer #2**

This manuscript assessing the legitimacy, ownership and embedded-ness acquired by the Quality Care Network in four separate countries and as an international entity is an ambitious and important project. My impression is that this could contribute to the global knowledge base through publication, however, some improvements should be made first. Details can be found in the uploaded document.

Author’s response: Thanks very much for your thorough review. We agree it could contribute to the global knowledge base and have made a number of revisions in response to your useful suggestions as detailed below.

1.A broad summary of the areas chosen for improvement: first, an overview of the QCN structure and activities--what are the key agreements that make it happen, what are the roles and responsibilities of participants? What motivates participation in the QCN?

Author’s response: This is covered in detail in another paper in our proposed collection evaluating the QCN, on network emergence by Shawar *et al,* and also in our paper on network effectiveness (Djellouli *et al*). We have added four sentences at the end of the first paragraph of our introduction to summarize this, referencing these two papers:

“QCN comprises a global secretariat based at the World Health Organization and is led in each of the 11 member countries by directorates in Ministries of Health [ref Shawar *et al*]. In each member country the quality of care network is made up of individuals representing a range of institutions including non-governmental organizations, professional associations, hospitals and health facilities, and district, regional and national ministries of health [ref Shawar *et al*]. The participants in the network are motivated by improving quality of care – increasing effective coverage – of interventions to reduce maternal, neonatal and child health mortality in hospitals [ref Shawar *et al*]. The roles and responsibilities of these participants in the network have included developing national roadmaps toward improved quality of hospital-based care for mothers and newborns, developing and implementing initiatives to improve quality of care at individual facilities, and strengthening data collection and use on indicators related to quality of care at hospital, district and national levels [ref Shawar *et al*, Djellouli *et al*].

2.Similarly, summary statements about what was found in the other papers about QCN and how they relate to this analysis--which of the case study countries were found to have effective QCN for example, and did that finding correspond to high legitimacy in this analysis?

Author’s response: In our discussion section we refer to key findings from our other papers including those on network effectiveness (Djellouli *et al*) and network emergence (Shawar *et al*) and how they relate to our findings in this paper:

“We did not find large differences in perceived legitimacy and government ownership of QCN work between our four case study countries despite large divergence in both the extent to which QCN emerged in each of them [10] and in how effective QCN was in each of them [11].”

We have added the following sentence suggesting further research on this, to the end of this paragraph of the discussion: “Further research that more explicitly examines the linkages between a network's emergence, effectiveness, and perceived legitimacy would be useful.

3.Another suggestion relates to greater attention to the working definitions of the key terms used in the analysis, beginning with "legitimacy." The chosen characteristic, an entity's ability "to exert power," does not seem wholly fitting.

Author’s response: The definition of legitimacy that we use, from Shiffman, 2015 (ref [3] of our paper” is “the right to exert power” so a legitimate right, i.e. an exertion of power that is just, rather than an ability to exert power (which could be exerted unjustly, i.e. illegitimately). We have added text below this, also in response to other comments, to clarify, so that it now reads:

“In this paper, legitimacy is understood as ‘the right to exert power’[3]. This right can be understood both in a normative and empirical sense. The former, from the perspective of democratic theory questions if an actor has a right to exert power, i.e., is QCN, which includes the global secretariat, as well as national and local actors, representative of constituent interests and/or historically effective in meeting those interests? The latter examines an actor’s perceived right to exert power from the perspective of a particular actor - in our case in this paper the ministry of health department that leads the network in country. For example, do domestic actors perceive the engagement and influence of QCN—especially of those actors that are external to the country (i.e., global secretariat and representatives from other involved QCN countries)—to be permissible?”

4. The figure depicting knowledge exchange with the differently weighted arrows could benefit from a re-work with more detailed explanation.

Author’s response: We have reworked Figure 1 – both the top part with the arrows in response to this comment, and the bottom part in response to other comments below. Please see our response to point 6 below.

5. Overall, more examples to support the statements would support the piece and its conclusions better.

Author’s response: We have provided a few examples under the sub heading collective decisions, for example, how the network is the reflection of collective decision according to one of the global respondents. We have also added more examples in this section to support the statement - please see our response to point 27 below.

6. It seems the authors have created a new framework by adapting three for this application--another figure expounding the adapted framework would support the conclusion that the framework could be used in other applications and could help readers digest the study.

Author’s response: We have now made Figure 1 much clearer including labelling the top part A) and the bottom part B). We have clearly explained how we have used the three frameworks (models) to make our own framework as depicted in this figure. We don’t think another figure is necessary as this figure contains our framework, adapted from the three published frameworks (models) we draw on. We have added a detailed caption for Figure 1 under the Figure 1 title:

“**A)** Depiction of interactions between institutions (actors) comprising the quality of care network (QCN) at global level (left) and national level (right). Interactions include political, normative and cognitive interactions between institutions as defined by Sanderink and Nasiritousi [21]. The width of the arrows indicates the strength of the interaction and is illustrative only and analyzed qualitatively. The width of the arrow depends on the variables in part B of the figure. **B)** We draw on work on individual, organizational and system capacities [22, 23] and models by Nasiritousi and Faber [13] (purple text) and Tallberg and Zurn [9] (orange text) on sources of institutional legitimacy to describe factors influencing the strength of interactions between institutions. These are depicted as characteristics of organizations (center of the diamond), organizational culture (next layer of the diamond) and wider culture and political stability surrounding the organization (outer diamond).”

7. Finally, another round of proofreading should be done. Some of the corrections are noted in the attached file.

Author’s response: Thank you for your careful review of our paper. Three of us have proofread the paper again before resubmission, and we have addressed all of the issues you raise in your annotations to the manuscript as described below and as seen as tracked changes to the manuscript.

8. Again, this work is appreciated as an uncommon attempt to make sense of how multi-country networks convened by global level actors achieve influence or fail to do so--its publication would be a contribution to global health practice. Best of luck to the authors for a successful round of edits.

Author’s response: Thank you very much. We hope we have addressed all of your comments and those of the other reviewer in our revision.

**Specific comments as annotations on the PDF of the paper by Reviewer 2 follow from here:**

9. P 2 L 28 – 29 “This requires legitimacy—a network’s right to exert power within national contexts. This is reflected, for example, in a government’s buy-in and perceived ownership of the work of the network”, Tricky ideas – the network could not have a right to exert power in a country unless there were policies and laws to support that. Perhaps soften this to “have influence”

Author’s response: From the perspective we adopt in the paper, that of the national government Ministry of Health quality departments that run the network in each country there is a right to exert power via the legal mandate the government has to govern.

10. P 3 L 56 At this point elucidate the QCN structure—references to country and level and global level follow, but it is unclear how these are structured—key to know when then considering the concept of legitimacy. Who drives action, what are the agreements, the incentives/motivation, and the roles and responsibilities.

Author’s response: As detailed in our response to point 1 above we have now summarized this immediately above at the end of the preceding paragraph.

11. P 3 L 59 – 60 “Each member country therefore must recognise the legitimacy of the network and take sufficient ownership of the required policy and management activities”, who requires the activities and how do participating countries indicate opting in

Author’s response: Here we are referring to policy and management activities required to deliver the strategic objectives of the network. We have clarified this by editing this sentence accordingly to: “Each member country therefore must recognise the legitimacy of the network and take sufficient ownership of policy and management activities required to deliver the strategic objectives of the network [ref Djellouli *et al*]”.

By becoming members of the network participating countries have opted in.

12. P 3 L 60 “In this paper, legitimacy is understood as the right to exert power”, So, is the question: do the country chapters of the Quality network have the right to exert power in their countries? or the international quality network? If so, who is that actor? A secretariat? Members from all participating countries? This could be clarified—country ownership vs outside body exerting power seem different

Author’s response: The country chapters of the quality of care network have the right to exert power in their countries as they are led by the ministry of health which has a legal mandate as part of the government (please see response to point 9 above). In newly added text (also see response to point 3 above) we clarify that the right of other external actors to exert power (their legitimacy) is an empirical question:

“In this paper, legitimacy is understood as ‘the right to exert power’[3]. This right can be understood both in a normative and empirical sense. The former, from the perspective of democratic theory questions if an actor has a right to exert power, i.e., is QCN, which includes the global secretariat, as well as national and local actors, representative of constituent interests and/or historically effective in meeting those interests? The latter examines an actor’s perceived right to exert power from the perspective of a particular actor - in our case in this paper the ministry of health department that leads the network in country. For example, do domestic actors perceive the engagement and influence of QCN—especially of those actors that are external to the country (i.e., global secretariat and representatives from other involved QCN countries)—to be permissible?”

13. P 3 L 64 “The latter (empirical sense) examines an actor’s perceived right to exert power from the perspective of a Particular actor”, who is the actor in this case

Author’s response: government MoH departments leading QCN teams (as per response to points 9 above)

14. P 3 L 68 “found an organisations performance”, apostrophe s

Author’s response: Thanks for spotting this, we have added the apostrophe.

15. P 5 L 96 – 97 “an iterative series of interviews of key stakeholders”, how were they chosen

Author’s response: Thanks so much! We have added a few lines to clarify how the key stakeholders were chosen.

16. P 5 L 97 “iterative series …………….document review”, what kind of docs? issued by?

Author’s response: We have pulled out the relative information from the supplementary method section and added here. Thanks!

17. P 5 L 113-114, “history of work on quality improvement in each country by the institutions involved in QCN relates to observed legitimacy, ownership and embeddedness” P 5-6, L 117-120, “We focused on three of these interactions, political, normative and cognitive (Figure 1) to investigate the legitimacy and ownership of the work of the network in each case study country, looking at the extent to which different organisations involved in the network work together across these three dimensions”, is legitimacy distinct from ownership? if so, how. I flow ownership, where does the legitimacy come from- another way clarifying structure and mode of operation of QCN will be helpful

Author’s response:	We understand legitimacy to be a pre-requisite of ownership, and following Vanyoro et al [9], who define ownership of research, we define ownership of implementation of the QCN here as: “the process whereby co-production enables health system actors (from policymakers to service users) to determine and influence [implementation] agendas with direct engagement with the [implementation] process itself” We have added this definition after this sentence, here:

“Consequent to QCN’s legitimacy we also investigate the ownership and direction of strategies adopted in each country, and how embedded the work of the network is in the health system (routine care) of each country. Following Vanyoro et al [9], who define ownership of research, we define ownership of implementation of the QCN here as: “the process whereby co-production enables health system actors (from policymakers to service users) to determine and influence [implementation] agendas with direct engagement with the [implementation] process itself”

18. P 8 Figure 1, Does the weight of the arrow mean something? If so, please describe—i see below— but that explanation still leaves something to be desired—is 1 pt of wieght for each additional human resource for example? How did you quantify to decide on the weights—or it is estimation for illustration only? I suggest reformatting this Figure to make it more clear. The title implies that drivers of legitimacy will be illustrated. It looks more like matrix of interaction, though the type of interactions is not clear

Author’s response: Thank you, we have revised the figure and added a caption to clearly explain it. The weights of the arrows are illustrative only, not quantitative. Please see our response to point 6 above. We have added a detailed caption for Figure 1 under the Figure 1 title:

“**A)** Depiction of interactions between institutions (actors) comprising the quality of care network (QCN) at global level (left) and national level (right). Interactions include political, normative and cognitive interactions between institutions as defined by Sanderink and Nasiritousi [5]. The width of the arrows indicates the strength of the interaction and is illustrative only and analyzed qualitatively. The width of the arrow depends on the variables in part B of the figure. **B)** We draw on work on individual, organizational and system capacities [16, 17] and models by Nasiritousi and Faber [8] (purple text) and Tallberg and Zurn [4] (orange text) on sources of institutional legitimacy to describe factors influencing the strength of interactions between institutions. These are depicted as characteristics of organizations (center of the diamond), organizational culture (next layer of the diamond) and wider culture and political stability surrounding the organization (outer diamond).”

19. P 8 F 1 Tallberg & Zurn, What is this figure?

Author’s response: Tallberg & Zurn is one of the frameworks (models) we draw on. Please see the newly added detailed caption for Figure 1 in the response above.

20. P 9 L 147-149 “We compared and contrasted these interactions in a cross-case synthesis to determine salient features of these institutional interactions in order

to evaluate the legitimacy of the network and ownership and embeddedness of the work of the network”, Some kind of working definition for ownership and embededness should be offered

Author’s response: Please see our response to point 17 above for the definition of ownership we have now added. For embeddedness, we have clarified in this sentence by adding “(integrated)”, we hope this is now clear:

“and how embedded (integrated) the work of the network is in the health system (routine care) of each country”

21. P10, L 181-182 “In addition to QIS, there were several development partners that have long worked on quality improvement in the country, including WHO, UNICEF, USAID and Save the Children. UNICEF worked in partnership with the Bill and Melinda Gates Foundation and the Ministry of Health (MoH) since 2015 to demonstrate a model of quality of care to scale up at national level via its Every Mother, Every Newborn (EMEN) pilot project in Kurigram (one of the northern districts in Bangladesh) [20]. Save the Children is a key implementer of USAID’s Mamoni Maternal, newborn and child health strengthening project (MNCSP), a flagship activity to support the Bangladeshi Maternal and Newborn Health program, started in 2018 [21]. These actors collectively engaged in the establishment and implementation of QCN activities”, Why did they do this? In order to hand over these MNH projects, keep them going, or why? And Did they do this independent of the Government but with their acceptance?

Author’s response: “......because of their long history of working on maternal and newborn health including quality of care. They had implemented the QCN activities independently but with the government’s acceptance as well as along with the government and connected to their previous work”, we have added the above lines, Thanks! We also have cited Shawer et al (reference10), another paper of our work, where the history and role of all organizations is described in detail.

22. P 12 L 216-217 “…..universal health coverage (UHC) and work done on HIV to trying to reduce mother-to-child transmission”, “HIV trying to reduce” or “on HIV to reduce mother”

Author’s response: “..HIV trying to reduce..”. Corrected, thank you.

23. P 12 L 221-222 “………….which formed a coordinating body (TWG) in charge of……..”, “a coordinating body, the Technical Working Group (TWG) in charge. . .”

Author’s response: Corrected, thank you.

24. P 12 L 230-232 “Cowater where PACHA was playing both the role of technical and implementing partner and …………………….. CHAI, …………… and EGPAF assisted to review the roadmap”, “Cowater, where PACHA” These are abbreviations for their full names—spell out The Clinton Health Access Initiative and Elisabeth Glaser Pediatric ADIS Foundation

Author’s response: Thanks, we have spelt out these acronyms

25. P 13 L 255 “UNICEF and UNFPA also supported other organisations (CBOs) to…….”, Say what this is before using the abbreviation

Author’s response: Thanks, CBOs are community-based organisations – we have added this to the text.

26. P 15 L 279 Table 1 “Political Interaction – collective decision”, such as?

Author’s response: These are described in the narrative text explaining the results shown in Table 1.

27. P 16 L 290 “Collective decisions, evidenced by a Memorandum of Understanding (MoU) or joint statement for example, should be artifacts of any multi-stakeholder network………”, Would an MOU be an example of a collective decision? Seems more like collaboration. This section would benefit from more detail—how many kinds of collective decisions were observed, did more collective decision making correlate with increased legitimacy or the other metrics?

Author’s response:	Thanks for your comment. We think MoU captures the collective decision(s) on paper, both parties agreed and sign on it. We have added more examples of collective decisions that we observed “In addition, the formation or reformation of TWG at the national level was an example of collective decision making. Development of QCN roadmap, preparation of national strategy, establishment of standards – were the reflection of joint statements or collective decisions”. Collective decision making led by or involving the ministry of health quality departments that lead the work of the network in each country reflects legitimacy of the network from the perspective of the ministry of health.

28. P 16 L 296 “At the national level, partners of all case countries working for QCN implementation, including those in the private sector, have contracts or longstanding bilateral arrangements with the MoH and with each other”, What does this mean? Is all setting up the agreements and monitoring? or is there clinical and service delivery work?

Author’s response: Thanks, we mean mostly setting up the agreements and monitoring here. Clinical and service delivery work was in a few cases but in those cases the organization assisted with providing human resources. We have added “(primarily setting up agreements and monitoring)” after “working for QCN implementation” to clarify what it means.

29. P 19 L 364 “Funding or sharing resources at the national and local levels is catalytic’, catalytic to what?

Author’s response: to quality improvement activities. Thanks, have added.

30. P 19 L 375 “Except human resources support, partners also provided logistics and financial support”, Do you mean “aside from” as opposed to “except” ?

Author’s response: Yes, corrected to “Aside from”, thanks

31. P 20 L 382 “In Uganda, partners pool their resources at the national level for QCN activities but previous funding experiences and prevailing implementing partner working arrangements with MOH, including tight timelines and targets, led funders to prefer directly funding their own QI initiatives ……….”, It seems important to say how this decision to pool resources for QCN was reached. Did MOH request it of partners or did the GLobal QCN team request it?

Author’s response: “This decision was taken by most funding partners, with the agreement of MoH” - we have added this sentence to clarify this. Previously most funders would put funds in a central pool for MOH to use - so they were working through MOH and not directly funding activities. However there were some challenges around allocation, disbursement and accountability. Partners did not want to stop their MNCH work; however, they also still needed value for money and to avoid financial loss and reduce bureaucratic delays associated with working through MOH. Through their platforms the partners voiced these concerns with the new proposed approach - to still fund MOH priority areas but directly fund activities and report to MOH on progress. MOH allowed their propositions, and many partners adopted this new approach.

32. P 20 L 396-398 “This suggests that the organizational and political culture of the MoH –their willingness to allow devolution or flexibility of funding– influences allocation of resources for QCN activities and reflects the extent to which the local level of QCN implementation was seen as legitimate by governments in each country”, are you sure this reflects legitimacy and not simply contextual financial policies?

Author’s response: We use the word “suggests”. This is based on the examples in this paragraph relating specifically to funding of QCN local activities rather than contextual financial policies in general.

33. P 21 L 409 “However, all agreed that network activities gave them more impetus to act and be accountable at global level”, Were they asked this question in a directed way like this or did they offer this statement? Were there examples?

Author’s response: Thanks for raising the point. They were asked whether the network adds value (please see the topic guide in supplement file), they agreed on this issue that the network activities gave them more impetus to act and be accountable at global level.

34. P 21 L 410 “All the actors sought synergy though they didn’t always experience synergy in terms of joint working on implementation and achievement of goals”, How did they seek synergy? Or did they desire synergy but not necessarily seek it directly?

Author’s response: Thanks, yes desired is more accurate – we have changed “sought” to “desired”.

35. P 23 L 469-470 “Overall, the lack of transfer of ideas and concepts between institutions and countries involved in QCN illustrated limits to the network, and the embedding of QCN work in government health systems”, Were there any ideas offered in the interviews for more suitable sharing methods or platforms?

Author’s response: We have checked our data and there were not any ideas offered for more suitable sharing methods or platforms. It may be that methods of sharing or platforms is not the main issue here, but rather (lack of) capacity or time and motivation to share ideas between institutions and countries.

36. P 26 L 499-500 “However, the webinars were mostly “DP centric” as government stakeholders hardly attended. A national respondent in Bangladesh noted”, what is DP—say [Development Partner] after DP and then it might good to interpret the comment— that the country leadership who are meant to benefit were not very engaged, or whatever you believe it to mean.

Author’s response: Thanks, we have revised to: ““DP [Development partner] centric” as country leadership didn’t have time to engage with them”

37. P 30 L 599-600 “the periphery of the network was found to be weaker, have less power, and be less networked and coordinated than the central level”, These are finding reported in another paper? I don’t think that the periphery is reported on in this paper

Author’s response: Yes these are findings from our stakeholder network analysis, already referenced here (reference [13] Mukinda et al)

38. P 30 L 608 “these finding”, findings

Author’s response: Thanks, corrected

39. P 30 L 609-610 “whilst the network was strong at global and national levels and useful for advocacy and sustaining the policy-profile of QCN objectives it did not often extend to influence day to day changes in practice at facility level”, practice at facility level and evaluation it is not really mentioned at all in this paper

Author’s response: Yes, you are correct. We have now cited our paper on network effectiveness [Djellouli *et* al] here.

40. P 30 L 610-611 “Organisational culture, and beliefs of network actors about the purpose of QCN may also have influenced its legitimacy, and consequent reach, at the local level”, Which organization’s culture? Is this statement supported by this study?

Author’s response: Organizational culture is included as a factor in this study (Figure 1 and results) though it’s influence, including at the local level is covered more in our study on individual, organisational and systems capacities [Tesfa *et al*] which we have now referenced here.

41. P 31 L 630-631 “We hope this framework may be useful in characterising the legitimacy of other implementation focused networks and the institutional interactions involved”, If this is an intention of this paper, a figure laying out your adapted framework would be useful

Author’s response: Please see our response to point 6 above. We don’t think another figure is necessary as Figure 1 (now revised as per response to point 6 above) contains our framework, adapted from the three published frameworks (models) we draw on.

42. P 32 L 659-661 “We did not find large differences in perceived legitimacy and government ownership of QCN work between our four case study countries despite large divergence in both the extent to which QCN emerged in each of them”, A summary of the findings of these previous studies of QCN would be very helpful early in this paper—which of the case study countries had high effectiveness of QCN etc.

Author’s response: Thank you. We have added two sentences summarising our studies on QCN emergence and QCN effectiveness respectively, at the end of our new additions to the first paragraph of the introduction:
“Our work on the emergence of QCN in four countries found that it emerged quickly and most robustly in Bangladesh, followed by Ethiopia, then Uganda, and Malawi [ref Shawar et al]. Our work on the effectiveness of QCN found that "Global and national leadership elements of QCN have been most effective to date, with action, learning and accountability more challenging, partner or donor dependent, remaining to be scaled-up, and pandemic-disrupted" [Djellouli et al].”

43. P 33 L 676 “increase perceived legitimacy, and reach, or the network”, of

Author’s response: Thanks, corrected to “of”

Yours sincerely,

Kohenour Akter

on behalf of the QCN Evaluation Group.
